# Knee Iliotibial Band Z-Plasty Lengthening and Bursectomy Technique

**DOI:** 10.1016/j.eats.2022.03.026

**Published:** 2022-07-14

**Authors:** Alex Vaisman, Rodrigo Guiloff, Domingo Andreani

**Affiliations:** a Orthopaedic Department, Facultad de Medicina Clínica Alemana, Universidad del Desarrollo, Santiago, Chile; b Clínica Alemana Santiago, Alemana Sport, Santiago, Chile

## Abstract

Multiple surgical techniques have been described to treat refractory iliotibial band syndrome. However, there is lacking evidence demonstrating superiority of one technique over the other and limited audiovisual resources. Most surgical procedures aim to release the iliotibial band; nevertheless, few focus on reducing concomitant inflammation. The present article illustrates a Z-plasty lengthening technique associated with local bursectomy for treating iliotibial band syndrome refractory to conservative treatment.

Iliotibial band syndrome (ITBS) is characterized by pain in the lateral aspect of the knee that increases with physical activity and decreases with rest.^1^ It is considered a sports-related overuse injury, presenting more frequently in athletes such as runners, cyclists, and hikers.^1^, ^2^, ^3^, ^4^, ^5^, ^6^, ^7^, ^8^, ^9^

The pathophysiologic mechanism that originates the ITBS is not entirely elucidated.^2^ Two main theories have been reported: one focuses on the mechanical friction between the iliotibial band (ITB) and the lateral femoral epicondyle; the other centers on the inflammation of the underlying bursal tissue.^7^^,^^8^^,^^10^^,^^11^ Both concepts should be addressed to achieve proper management of this pathology.

ITBS is mainly treated conservatively, and is based on rest, ice, and nonsteroidal anti-inflammatory drugs associated with physical therapy focusing on stretching the ITB and thigh lateral structures, strengthening hip abductors, neuromuscular control, and improving function.^1^^,^^3^^,^^5^^,^^7^^,^^11^, ^12^, ^13^ Local steroids injections can be used when other conservative measures have failed to confer clinical improvement.^1^^,^^4^^,^^6^^,^^10^^,^^13^ More than 90% of the patients respond favorably to these nonoperative measures^11^^,^^14^ and return to sports after 3 to 4 months.^9^^,^^12^^,^^15^ In those cases refractory to conservative treatment (usually after 3-6 months), surgical intervention is advocated.^1^, ^2^, ^3^, ^4^, ^5^

Multiple surgical options to treat ITBS have been described, including open and arthroscopic techniques (Table 1).^2^ However, these techniques aim either to release the ITB or to reduce inflammation by excision of underlying bursal tissue without considering a multifactorial etiology. The present article presents a technique that combines ITB Z-plasty lengthening associated with local bursectomy, adapted from Barber et al.^3^^,^^8^ and Hariri et al.^11^ The objective of the present study is to illustrate this technique.

**Table 1:** Surgical Options to Treat Refractory ITBS

Author	Year	Type of Study	n	Surgical Technique(s)	Return to Sports	Clinical Postoperative Results	Complication(s)
Noble^4^	1979	Case series	9	Posterior ITB triangle resection	88.8% (running) at 2-16 months	–	Recurrent pain (1)
Martenset al.^5^	1989	Case series	19	Posterior ITB triangle resection	100% same level (football, running, and cycling) at 7 weeks	100% satisfied	Hematoma with surgical revision (1)
Holmes et al.^6^	1993	Case series	4	Percutaneous release	25% same level (cycling)	– 71.4% pain-free activity	Open surgical revision (3)
			21	Ellipse resection	81% same level (cycling) at 6-8 weeks		Hematoma (2), seromas (9), and surgical ellipse revision (1)
Drogsetet al.^7^	1999	Case series	45	Posterior ITB hemisection ± bursectomy	–	84.5% good-excellent subjective results	Wound infection (1), residual pain (20), knee weakness (2), and local effusion (1)
Richards et al.^8^	2003	Technical note	1	Arthroscopic exploration + Z-plasty lengthening	–	–	–
Sangkaew^17^	2006	Technical note	1	Mesh: multiple punctures adjacent to the epicondyle	–	Pain-free, return to occupational activity	–
Boothby et al.^3^	2007	Case series	8	Z-plasty lengthening	100% same level at 59-97 months	100% resolution of original lateral knee pain. Cincinnati: 82.9, Tegner: 4.4, Lysholm: 88.6, and IKDC∗: 2.6	None
Hariri et al.^11^	2009	Case series	11	Arthroscopic exploration + open bursectomy	72.3% same or higher level at 2 years	54.5% completely satisfied, 27.3% mostly satisfied. Tegner: 5, Lysholm: 94.1, and IKDC∗: 87.5	–
Michelset al.^12^	2009	Case series	35	Arthroscopic lateral gutter synovial recess resection	100% (running) at 3 months	97.1% good-excellent subjective results	Hematoma with surgical revision (1)
Cowden and Barber^16^	2014	Case report	1	Arthroscopic Kaplan fiber and lateral synovial recess resection	Same level at 4 weeks	Satisfied, pain-free at 4 weeks	None
Inoue et al.^19^	2017	Case series	31	Split-thickness lengthening	100% (competition) at 5.8 weeks	No extensor and flexor muscle strengths differences between affected and healthy sides at 2 months	None
Walbron et al.^9^	2018	Technical note	14	Release from Gerdy’s tubercle	Same level at 4 months	85.7% satisfaction rate. Tegner: 6 and Lysholm: 93	Deep venous thromboses (2)
Dart et al.^18^	2021	Technical note	1	Z-plasty lengthening	Same level (time not described)	–	None

NOTE. Postoperative clinical scores are given in mean values. “–” indicates not clearly described.

IKDC, International Knee Documentation Committee; ITBS, iliotibial band syndrome.

∗ Might have used different scoring systems.

## Surgical Technique (With Video Illustration)

A narrated step-by-step demonstration of the knee ITB Z-plasty lengthening and bursectomy technique may be reviewed in Video 1. The patient is positioned supine, and a knee diagnostic arthroscopy is performed through an anterolateral portal to rule out other possible concomitant pathology. The authors routinely use a tourniquet during the whole procedure.

**Fig 1:**
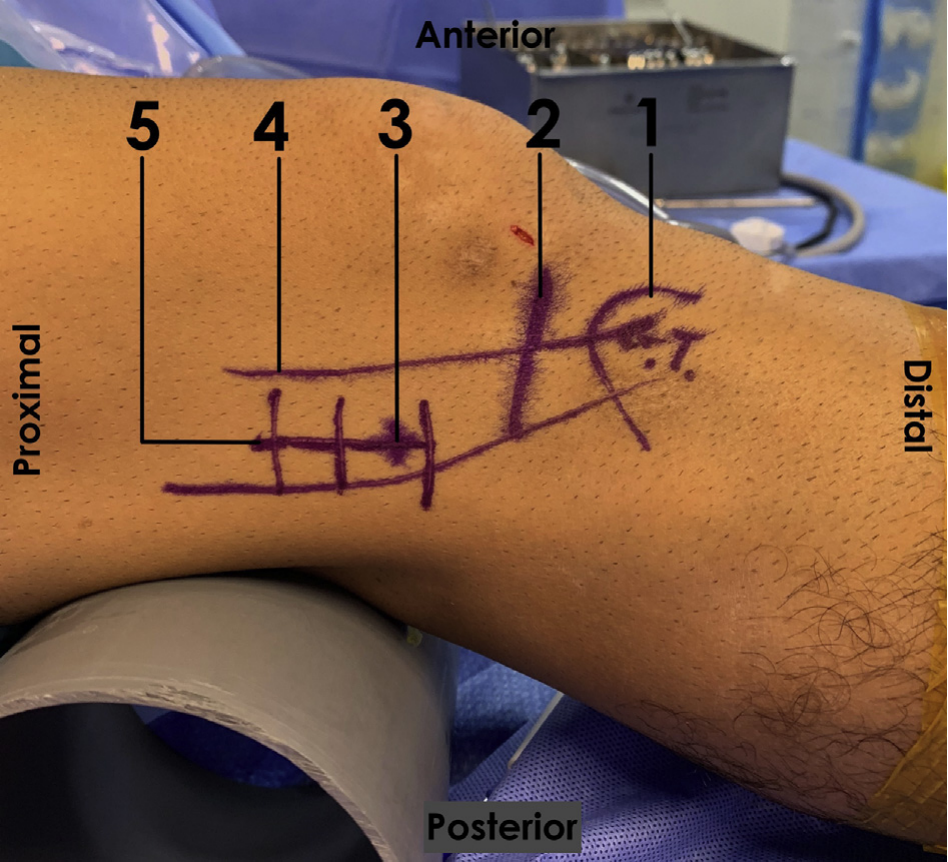
Surface anatomy landmarks. In a right 30° flexed knee, with the patient in a supine position, the surface anatomy landmarks are recognized, such as Gerdy’s tubercle (1), which corresponds to the distal insertion of the ITB and can be located by direct palpation. It is necessary to establish the joint line (2) and the lateral femoral epicondyle (3) location to trace the path of the ITB (4) proximally along the lateral face of the thigh. The surgical approach is created by a 4-cm lateral incision (5) along the ITB’s axis. (ITB, iliotibial band.)

After the arthroscopy, the knee is flexed at 30° (where the greatest tension between the ITB and the lateral epicondyle occurs^16^), and skin landmarks including Gerdy’s tubercle, the lateral femoral epicondyle, and the tibiofemoral joint line are marked (Fig 1).

**Fig 2:**
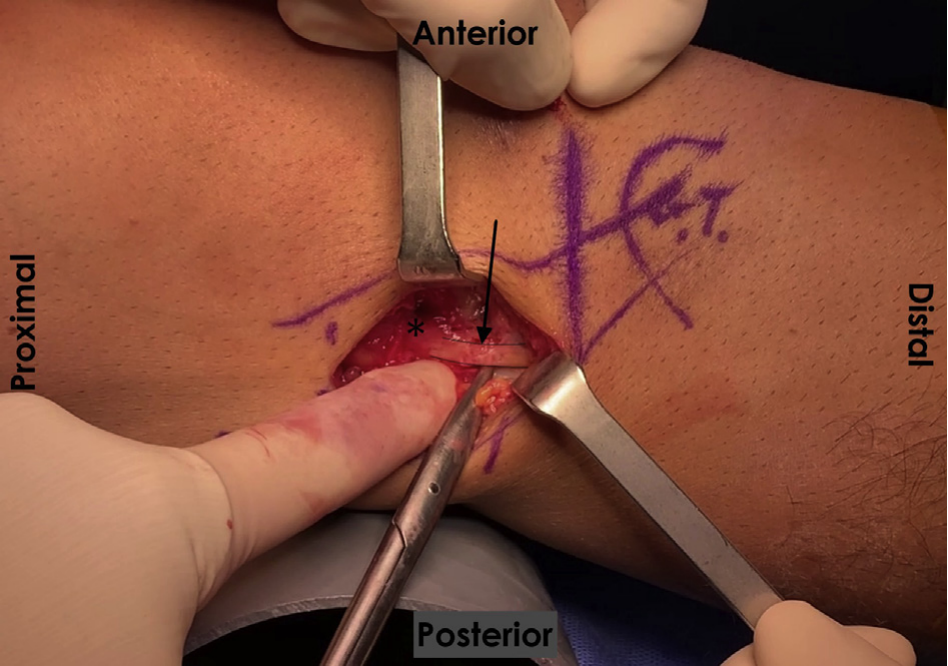
Lateral collateral ligament identification. In a right knee, via a lateral incision in the supine position with the knee flexed at 30°, the lateral collateral ligament (arrow), which lies underneath the ITB (which has been anteriorly reflected with a retractor, and not visible in this picture), must be identified and protected as it is the main structure at risk due to its proximity to the lateral epicondyle (asterisk). (ITB, iliotibial band.)

**Fig 3:**
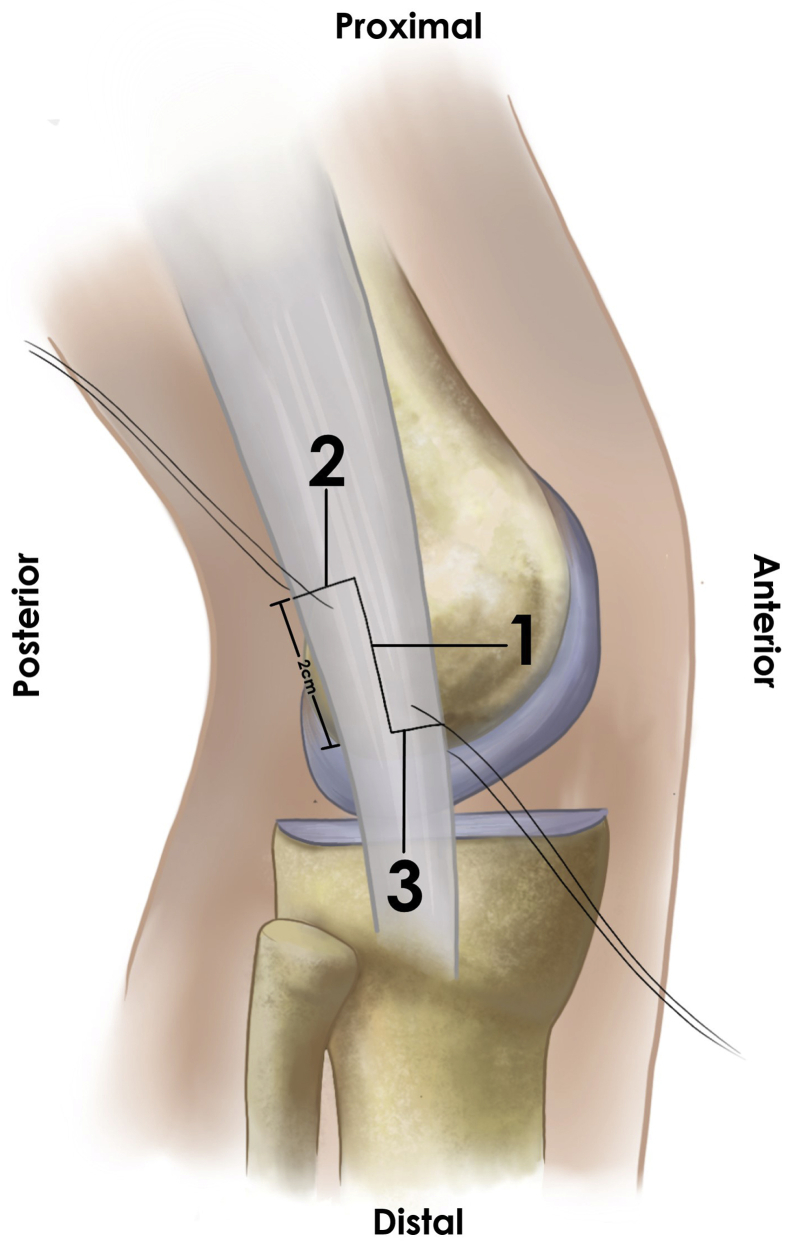
Preparing the ITB Z-plasty. Schematic representation of the ITB in a right knee. A 2-cm longitudinal line is drawn along the ITB’s central axis (1), with its center at the level of the lateral femoral epicondyle. At its proximal end, a perpendicular line is drawn towards the ITB’s posterior edge (2). Another perpendicular line is marked at its distal end towards the ITB’s anterior edge (3), completing the “Z” figure. The authors recommend making rein sutures on the “Z” arms to facilitate its mobilization. (ITB, iliotibial band.)

**Fig 4:**
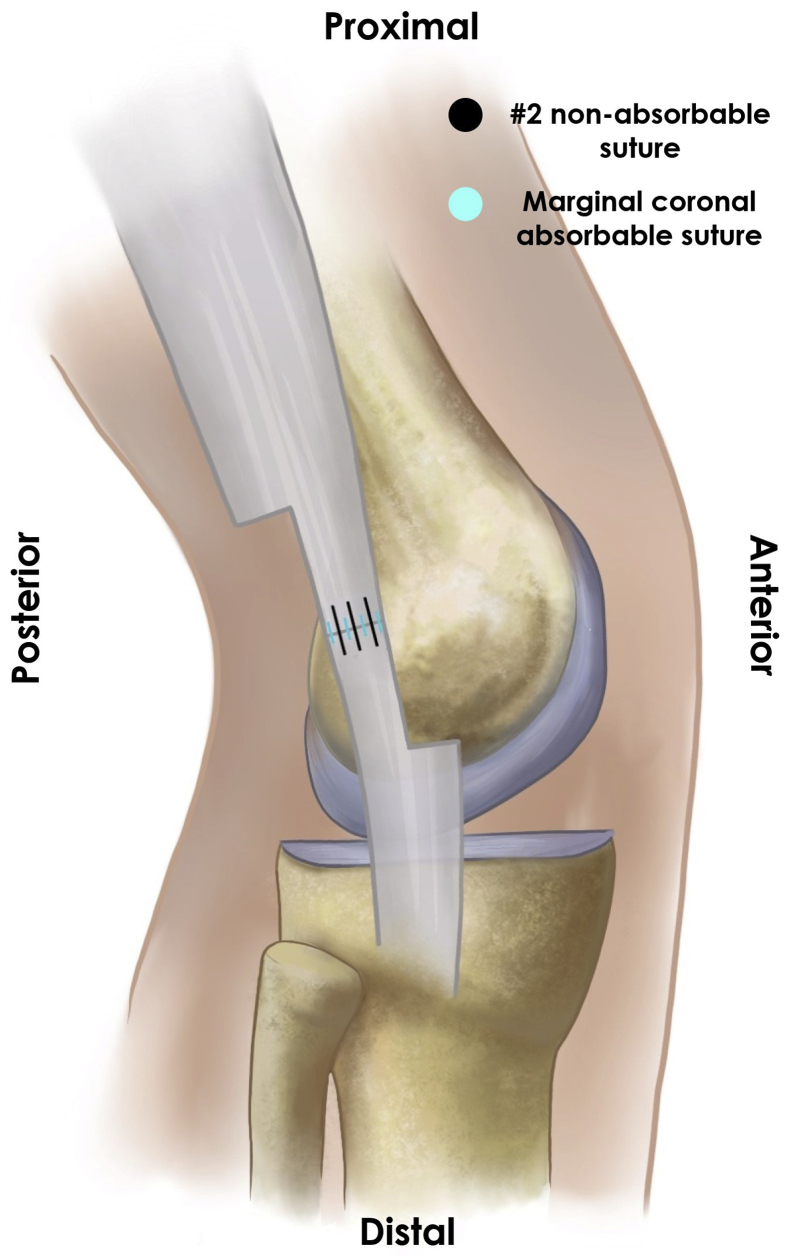
End-to-end ITB repair. Schematic representation of the ITB Z-plasty in a right knee. After a complete ITB section, both “Z” arms are attached in an end-to-end fashion by employing simple stitches with a #2 nonabsorbable suture (black), resulting in a 2-cm ITB lengthening. The Z-plasty lengthening is reinforced with marginal coronal absorbable sutures (light blue), considering not overtensioning the band. (ITB, iliotibial band.)

The surgical exposure is created by a 4-cm incision along the axis of the ITB, beginning approximately 2 cm proximal to the joint line. Dissection of the subcutaneous tissue should allow complete visualization of the ITB to perform a proper release of its anterior and posterior edges. Local fibrous adhesions should be released proximally and distally to isolate the band from deeper tissues. This allows an anterior mobilization of the ITB, exposing the underlying inflammatory bursal tissue, which should be excised. The authors recommend sending a bursal sample for pathology evaluation to confirm bursal inflammation. The knee lateral collateral ligament must be identified and protected before proceeding with the Z-plasty to avoid potential iatrogenic residual knee instability (Fig 2). Next, a 2-cm longitudinal line is drawn along the ITB’s central axis, with its center at the level of the lateral femoral epicondyle. A perpendicular line is drawn toward the ITB’s posterior edge at its proximal end. Another perpendicular line is marked at its distal end, this time toward the ITB’s anterior edge, which completes the “Z” figure. Before incising the band, the authors recommend making rein sutures on the “Z” arms to facilitate its mobilization (Fig 3). The “Z” figure is carefully cut using a number 23 scalpel, preventing any potential damage to the deeper structures. After a complete ITB section, both “Z” arms are attached in an end-to-end fashion by employing simple stitches with a number 2 high resistance nonabsorbable suture, resulting in a 2-cm ITB lengthening. The authors advocate for the use of nonabsorbable sutures, as absorbable sutures could lose tension before the ITB has healed, causing premature repair failure. The Z-plasty lengthening is reinforced with marginal coronal absorbable sutures; over-tensioning the band must be avoided (Fig 4). Once completed, stability must be tested by performing passive and full range of knee motion. Finally, a closure by layers is made. The authors recommend intradermal stitches for better aesthetic results. Table 2 summarizes the pearls and pitfalls of this procedure.

**Table 2:** Pearls and Pitfalls

Pearls
• Removal of adhesions below the band allows its correct manipulation
• Bursectomy: removing the inflamed bursal tissue may aid in alleviating symptoms
• Rein sutures before incising the band facilitate the manipulation of its ends
Pitfalls
• Failure to identify and protect the LCL: if injured, iatrogenic varus instability may be produced
• Overtensioning ITB end-to-end sutures: enough tension to close the band and secure its stability in a full range of motion should be applied, being careful not to overtighten it. Otherwise, ITBS symptoms may persist
• Absorbable sutures could lessen their tension before the ITB has healed, causing premature plasty failure: high resistance, non-absorbable sutures should be used to fix the Z-plasty to avoid this complication

ITB, iliotibial band; ITBS, iliotibial band syndrome; LCL, lateral collateral ligament.

### Postoperative Rehabilitation Protocol

The postoperative rehabilitation protocol includes immediate weight-bearing assisted by 2 crutches, range of motion as tolerated, and quadriceps isometric–strengthening exercises. Patients will usually no longer need crutches between 1 and 2 weeks after surgery. At 4 weeks, a nonimpact workout is started, with stationary biking and swimming, including balance recovery and close chain strengthening exercises. At 8 weeks, plyometric exercises are started, including jumps and pivoting drills. A progressive return to sports program begins once the patient has achieved appropriate lower extremity strength, range of motion, and proprioception. Patients are allowed to return to sports 12 weeks after surgery.

## Discussion

Multiple surgical techniques have been described to treat refractory ITBS, such as a posterior triangular resection of the ITB,^4^^,^^5^ elliptical resection,^6^ transverse sectioning of the posterior half,^7^ multiple punctures of the band, or the mesh technique,^17^ isolated bursectomy,^11^ digastric release from Gerdy’s tubercle,^9^ and the Z-plasty lengthening technique.^3^^,^^8^

Despite the broad range of surgical procedures, the Z-plasty lengthening and bursectomy technique appears as an attractive procedure that combines the treatment of 2 of the most accepted pathophysiologic mechanisms. On the one hand, it lengthens the ITB, decreasing the friction between the band and the lateral epicondyle. On the other hand, removing the inflammatory tissue, especially the bursae, reduces pain and inflammation. Due to its broader approach, this merging surgical technique should be a more appropriate method to treat refractory ITBS.

There are limited clinical data and audiovisual material demonstrating the Z-plasty technique. Recently, Dart et al.^18^ published a video on the Z-plasty lengthening technique. However, they only described one case with a more conservative rehabilitation protocol (knee brace and 50% weight-bearing for 6 weeks). Their video shows a side-to-side suture of the ITB, which may cause greater shear stress forces on the plasty and condition an early failure of the repair, as opposed to the end-to-end technique shown in the present article. This might be the reason for their need of a more conservative rehabilitation protocol. The present technique was adapted from the Z-plasty lengthening technique described by Barber et al.^3^ They showed a case series with full return to the previous activities. However, their validated patient-reported outcomes measurements (Tegner and Lysholm) were slightly lower than those reported by Hariri et al.^11^ This difference between functional results might be explained due to the open bursectomy performed by Hariri et al.,^11^ which was not performed in Barber’s case series, yet included in the present Technical Note.

In conclusion, this study describes the ITB Z-plasty lengthening technique with associated bursectomy for treating ITBS refractory to conservative treatment.

## Supplementary Data

Full ICMJE author disclosure forms

Video 1 Surgical demonstration of the knee ITB Z-plasty lengthening and bursectomy technique. The case presented is a male patient with right knee refractory ITBS. After a diagnostic arthroscopy through an anterolateral portal, the patient is positioned supine, and the knee is flexed at 30°. Next, skin landmarks are marked. The surgical exposure is created by a lateral 4-cm incision along the axis of the ITB, beginning approximately 2 cm proximal to the joint line. Fibrous adhesions are released to allow anterior mobilization of the ITB, exposing the underlying inflammatory bursal tissue, which should be excised. Next, the LCL is identified and protected, and the “Z” is marked along the ITB’s central axis, with its center at the level of the lateral femoral epicondyle. The authors recommend making rein sutures on the “Z” arms to facilitate its mobilization. After completing the “Z” ITB section, both “Z” arms are attached in an end-to-end fashion by employing simple stitches with high resistance non-absorbable sutures, resulting in a 2-cm ITB lengthening. The Z-plasty lengthening is reinforced with marginal coronal absorbable sutures; overtensioning the band must be avoided. Once completed, stability is tested, and closure by layers is made. (ITB, iliotibial band; ITBS, iliotibial band syndrome; LCL, lateral collateral ligament.)
